# Prolonged immune alteration following resolution of acute inflammation in humans

**DOI:** 10.1371/journal.pone.0186964

**Published:** 2017-10-26

**Authors:** Madhur P. Motwani, Justine Newson, Simon Kwong, Angela Richard-Loendt, Romain Colas, Jesmond Dalli, Derek W. Gilroy

**Affiliations:** 1 Centre for Clinical Pharmacology and Therapeutics, Division of Medicine, University College London, London, United Kingdom; 2 Division of Neuropathology and, Dept. of Neurodegenerative Disease, University College London Institute of Neurology, Queen Square, London, United Kingdom; 3 Lipid Mediator Unit, William Harvey Research Institute, Bart’s and the London School of Medicine, Queen Mary University of London, London, United Kingdom; University of Calgary, CANADA

## Abstract

Acute inflammation is an immediate response to infection and injury characterised by the influx of granulocytes followed by phagocytosing mononuclear phagocytes. Provided the antigen is cleared and the immune system of the host is fully functional, the acute inflammatory response will resolve. Until now it is considered that resolution then leads back to homeostasis, the physiological state tissues experienced before inflammation occurred. Using a human model of acute inflammation driven by intradermal UV killed *Escherichia coli*, we found that bacteria and granulocyte clearance as well as pro-inflammatory cytokine catabolism occurred by 72h. However, following a lag phase of about 4 days there was an increase in numbers of memory T cells and CD163^+^ macrophage at the post-resolution site up to day 17 as well as increased biosynthesis of cyclooxygenase-derived prostanoids and DHA-derived D series resolvins. Inhibiting post-resolution prostanoids using naproxen showed that numbers of tissue memory CD4 cells were under the endogenous control of PGE_2_, which exerts its suppressive effects on T cell proliferation *via* the EP4 receptor. In addition, we re-challenged the post-resolution site with a second injection of *E*. *coli*, which when compared to saline controls resulted in primarily a macrophage-driven response with comparatively fewer PMNs; the macrophage-dominated response was reversed by cyclooxygenase inhibition. Re-challenge experiments were also carried out in mice where we obtained similar results as in humans. Therefore, we report that acute inflammatory responses in both humans and rodents do not revert back to homeostasis, but trigger a hitherto unappreciated sequence of immunological events that dictate subsequent immune response to infection.

## Introduction

Following infection the acute inflammatory cascade is initiated with the specific aim of eradicating the pathogen [1]. Thereafter, a sequence of events takes place including catabolism of pro-inflammatory cytokines and chemokines [2,3], as well as inhibition of granulocyte recruitment to the affected site [4]. Of the infiltrated granulocytes the majority have been shown to die by apoptosis and are cleared by tissue-resident macrophages [5]. This entire process is relatively rapid occurring within 3–5 days.

According to current view, upon successful resolution the inflamed tissue returns back to homeostasis—the cellular and biochemical state present before inflammatory challenge. This view has been challenged by recent reports which show that resolution is not just the termination of innate immune response to infection/injury, but leads to cellular and biochemical events that can influence subsequent adaptive immune responses [6–9]. Indeed, we found that following resolution of acute peritonitis there was sustained infiltration of myeloid and lymphoid cells into the peritoneum that persisted for months [9]. We hypothesised that this post-resolution infiltrate bridged the gap between innate and adaptive immunity as depleting myeloid cells, for instance, during this phase blunted lymph node expansion. Moreover, a population of these infiltrated myeloid cells were retained in the peritoneum long-term and dictated the severity and longevity of subsequent innate immune-mediated responses to secondary inflammatory stimuli [9,10]. Some infections can also cause “immunological scarring”, such that despite effective clearance of the inciting stimulus, the site becomes chronically inflamed rather than reverting to homeostasis [11,12]. Taken together, these reports highlight that our current understanding of resolution should not be limited to defining it as just the end of innate immune–mediated responses to infection. Instead, once the clinical signs of inflammation have abated, the site of inflammation experiences a great deal of immunological activity occurring at the sub-clinical level that dictates the subsequent long-term physiological fate of tissues post injury.

Using a recently developed model of self-resolving acute inflammation in healthy human volunteers we sought to characterise the local immunological events that occur after resolution of the response. Injecting UV-killed *E*. *coli* (UVkEc) into the dermis triggered a robust neutrophilia and localised cytokine flare peaking at 4h. This was followed by neutrophil clearance alongside monocyte/macrophage infiltration. However, by day three resolution had occurred at least according to the classical pathological definition of granulocyte and antigen clearance as well as reversal of the cardinal signs of heat, redness, swelling and pain. Following resolution, we noted increased numbers of memory T cells, macrophage populations as well as robust lipid mediator biosynthesis, which we contend plays a key role in maintaining post-resolution homeostasis and mounting responses to secondary infection.

## Methods

### Ethics statement

Study approval was obtained from UCL Institutional Ethics Committee (Project ID: 5051/001). All volunteers provided written informed consent and study procedures were in accordance with the Helsinki Declaration of 1975, revised in 1983.

### Study participants

Healthy non-smoking male volunteers (18–50 years) were excluded if they had history of any chronic inflammatory disease, recent illness (<1 month), vaccination within the last three months, regularly took prescribed medication, or took any medication in preceding week. Volunteers with history of hypersensitivity to NSAIDs or gastrointestinal ulceration were also excluded. During the study period, volunteers were asked to refrain from alcohol and heavy exercise.

### Injection of UV-killed E. coli (UVkEc) and acquisition of inflammatory exudate

Acute inflammation was triggered by intradermal injection of 1.5 x 107 UV-killed *E*. *coli* bacteria suspended in 100 μl of saline in the forearm. Inflammation was allowed to progress for the duration of the time-point after which a suction blister was raised over the marked injection site, followed by immediate aspiration of inflammatory exudate. Inflammatory exudate was centrifuged to separate cells from supernatant. Cells were processed immediately for flow cytometry and the cell free exudate was frozen into aliquots at -80^0^ C and was analysed later for soluble mediators. The detailed description of the preparation of UVkEc, intradermal injection, suction blister technique, exudate collection and processing can be found in reference [13]. COX-inhibition studies were performed by orally administering 500mg enteric-coated naproxen tablets, twice daily.

### Flow cytometry

Blister cells were suspended in 100 μl of cell staining buffer (PBS with 5% FCS, 0.1% sodium azide) were incubated for 30 mins on ice with following antibodies: CD3: FITC, CD14: BV605, CD16: APC, CD19: FITC, CD56: FITC, CD62L: PE-Cy7, CD163: BV421, HLA-DR: BV510, CD45RO: BV785, CCR7: BV711 (all antibodies were obtained from Biolegend). Stained cell sample was washed to remove excess antibody and then fixed in 1% paraformaldehyde. Fixed sample was acquired on BD LSR Fortessa within 4h. Flow cytometry data was analysed by Flowjo software (Treestar Inc.). Flow cytometric gating strategy employed to identify cell populations is as described in reference [13].

### Multiplex ELISA

Human pro-inflammatory panel-1 mutiplex ELISA kit (Meso Scale Delivery, USA) was used to measure cytokines. The cell free blister exudate was diluted in appropriate assay diluent and the assay was performed as per manufacturer’s instructions.

### Immunohistochemistry

A skin punch biopsy (3 mm diameter) was obtained from the site of UVkEc injection at the specified time point. Baseline biopsy was obtained from forearm of a non-injected volunteer. Biopsies were immediately transferred to neutral buffered formalin for fixation. Fixed skin biopsies were embedded in paraffin wax. Four μm thickness sections were collected on the glass slides and stained for antibodies after unmasking of antigen. Following antibodies were used: Mouse anti- *E*. *coli* LPS antibody (Abcam), Mouse anti-CD163 antibody (Leica). Antibody stained slides were developed using 3′3 diaminobenzedine tetrachloride as the chromogen followed by counterstaining by haematoxylin for localization of nuclei. Appropriate controls were used throughout. Digital images of the stained slides were obtained on a Nanozoomer (Hamamatsu).

### Lipid mediator profiling

Blister exudates were placed in 2 volumes ice-cold methanol containing deuterium labelled PGE2 (d4-PGE2); d_4_-leukotriene B_4_, d_5_- lipoxin A_4_ and d_5_-resolvin D2 (500 pg each; Cayman Chemicals). These were then kept at -20 oC for 45 mins to allow for protein precipitation and lipid mediators were extracted using C-18 based Solid Phase Extraction as in reference [14]. Methyl formate fractions were brought to dryness using a TurboVap LP (Biotage) and products suspended in water-methanol (80:20 vol:vol) for Liquid Chromatography-tandem mass spectrometry (LC- MS/MS) based profiling. Here a Shimadzu LC-20AD HPLC and a Shimadzu SIL- 20AC autoinjector (Shimadzu, Kyoto, Japan), paired with a QTrap 5500 (ABSciex, Warrington, UK) were utilised and operated as described in reference [14]. To monitor each lipid mediator and deuterium labelled internal standard, a Multiple Reaction Monitoring (MRM) method was developed using parent ions and characteristic diagnostic ion fragments as in reference [14]. This was coupled to an Information Dependent Acquisition and an Enhanced Product Ion scan. Identification criteria included matching retention time (RT) to synthetic standards and at least six diagnostic ions in the MS-MS spectrum for each molecule. Calibration curves were obtained for each molecule using authentic compound mixtures and deuterium labelled lipid mediator at 0.78, 1.56, 3.12, 6.25, 12.5, 25, 50, 100, and 200 pg. Linear calibration curves were obtained for each lipid mediator, which gave r2 values of 0.98–0.99.

### Mononuclear/T cell interactions studies

Forty millilitres of blood was taken from healthy volunteers in heparin vacutainers and diluted 1:3 in hanks balanced salt solution (HBSS, Life technologies), and layered on Ficoll (GE Healthcare), spun at low acceleration. The peripheral blood mononuclear cells (PBMC) layer was carefully removed and washed in PBS. PBMC’s were incubated with anti-human CD3 beads (Miltenyi Biotec) and the flow through was plated and incubated with 1 μg/ml VZV (Virusys lot A1446100) for 4 h with or without PGE_2_ (Cayman Chemical, see legends for concentrations) or 25% blister fluid from day 17 of *E*. *coli* blister model. Cells were washed and incubated with CD3 cells with or without PGE_2_ at a ratio of 1:3 PBMC to CD3 cells respectively for 3 days. Proliferation was measured with anti-Ki67.

### Animal studies and drug dosing

Male C57Bl6/J mice (aged 8–10 weeks) were maintained in accordance with United Kingdom Home Office regulations (Project Licence number P69E3D849, Establishment Licence number X7069SDD). Male C57Bl6/J mice (15 in total) were maintained in an enriched environment and allowed water and food *ad libitum*. Peritonitis was induced by injecting sonicated 0.1 mg/mouse zymosan A (Sigma) intraperitoneally. While this type of inflammatory response is localised in nature and does not cause pain or distress, animals were nonetheless monitored daily. For re-challenge experiments 40000 CFU *Streptococcus pneumoniae*^*ova323-339*^/mouse were injected into the peritoneal cavity 14 days after zymosan injection. This secondary stimulus was allowed to progress for 4 h during which animals were constantly monitored with no clinical manifestation of pain or systemic inflammation observed during this time. *Streptococcus pneumoniae*^*ova323-339*^ was obtained from Prof Jeremy Brown, UCL. Animals were sacrificed by excess carbon dix oxide inhalation. For cyclooxygenase inhibition studies, naproxen was dosed at 30mg/kg/day starting from day 7 after zymosan injection.

## Results

### UV-killed *E*. *coli* triggers acute resolving inflammation in healthy humans

**Fig 1:**
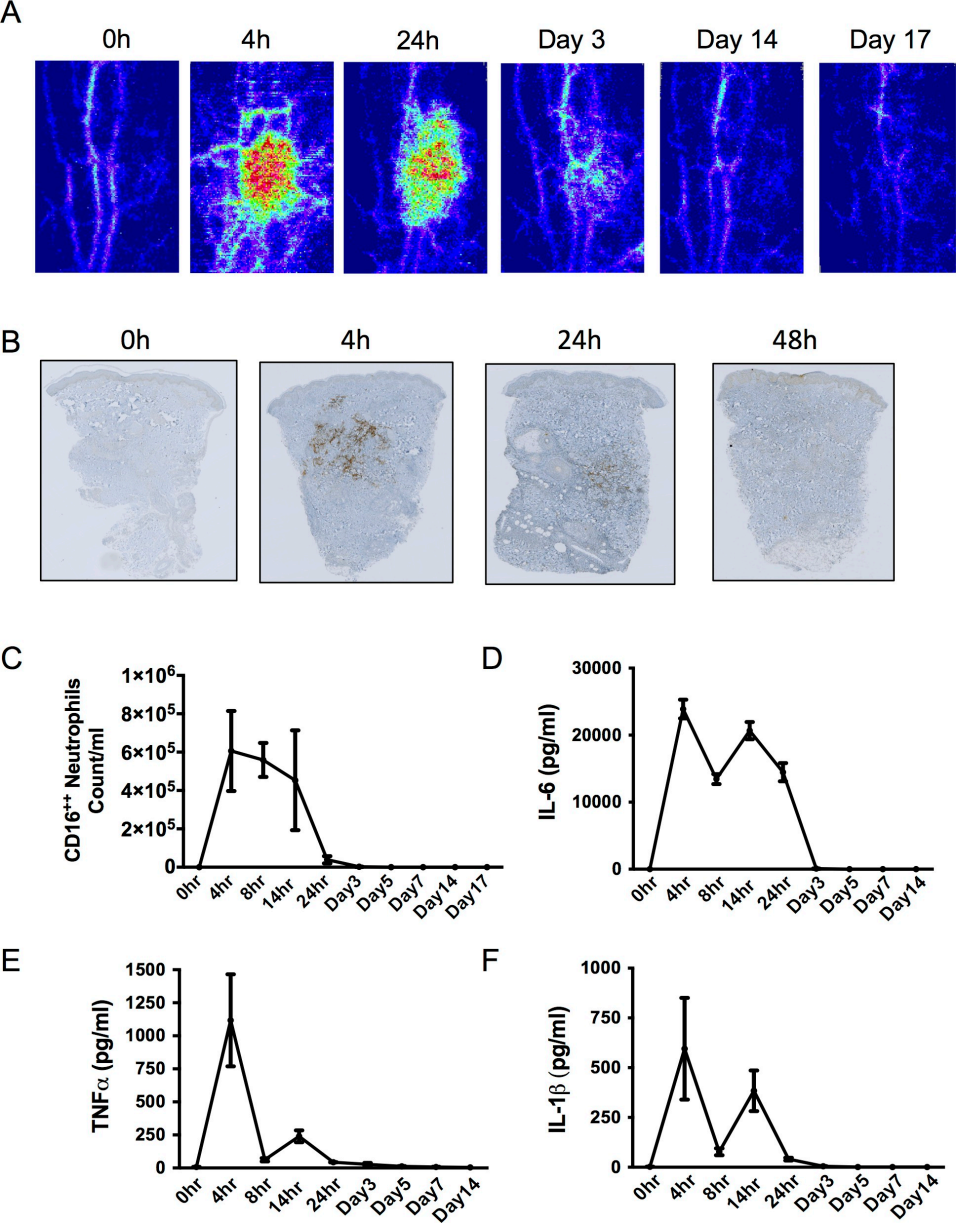
Acute inflammatory response to intradermal UVkEc injection. Acute inflammation was triggered in the ventral aspect of forearm of healthy volunteers by the intradermal injection of 1.5 x 10^7^ UV-killed *E*. *coli* (UVKEc) suspended in 100 μl of sterile saline. Vascular hyperaemia at the site was assessed by laser Doppler imager and the representative flux images after a specified time point are shown here **(A)**. A 3 mm skin punch biopsy was taken from the inflamed site under local anaesthesia at the specified interval and formalin fixed paraffin embedded (FFPE) skin sections were probed by immunohistochemistry for *E*. *coli* LPS. The representative sections are shown here **(B)**. A suction blister was raised over the inflamed site at the specified interval to collect the inflammatory exudate. Exudate was centrifuged to separate cells from the supernatant. The immune cell subsets were identified by polychromatic flow cytometry. The total count/ml of neutrophils in the inflammatory exudate at specified interval is shown here **(C)**. IL-6, TNFα and IL1β in the cell free exudate were measured using multiplex ELISA and their concentrations in the exudate at the specified intervals are shown here **(D-F, respectively)**. n = 3 for each time point. Data presented as mean ± SEM.

The intradermal injection of 1.5x10^7^ UVkEc into the forearm of healthy male volunteers caused an increase in microvascular hyperaemia around the site of injection that persisted from 4 h to 24 h and had resolved by day 3, **Fig 1A**. Long before the resolution of the microvascular responses was the clearance of the UVkEc, which was detectable at the injection site at the peak of the acute immune response (4 h), but was largely cleared by 24 h being undetectable at 48 h, **Fig 1B**. Finally, using negative pressure suction blisters to acquire cells and inflammatory mediators from the inflamed site we found that PMN numbers **(Fig 1C)** as well as typical pro-inflammatory cytokine levels (IL-6, TNFα and IL-1β) peaked at 4 h, but were undetectable by 24 h, **Fig 1D–1F**. Thus, the injection of UVkEc bacteria into the forearm of healthy volunteers generated a transient acute inflammatory response that resolved by 72 h.

### Post-resolution infiltration of lymphocytes and CD163^+^ macrophages

**Fig 2:**
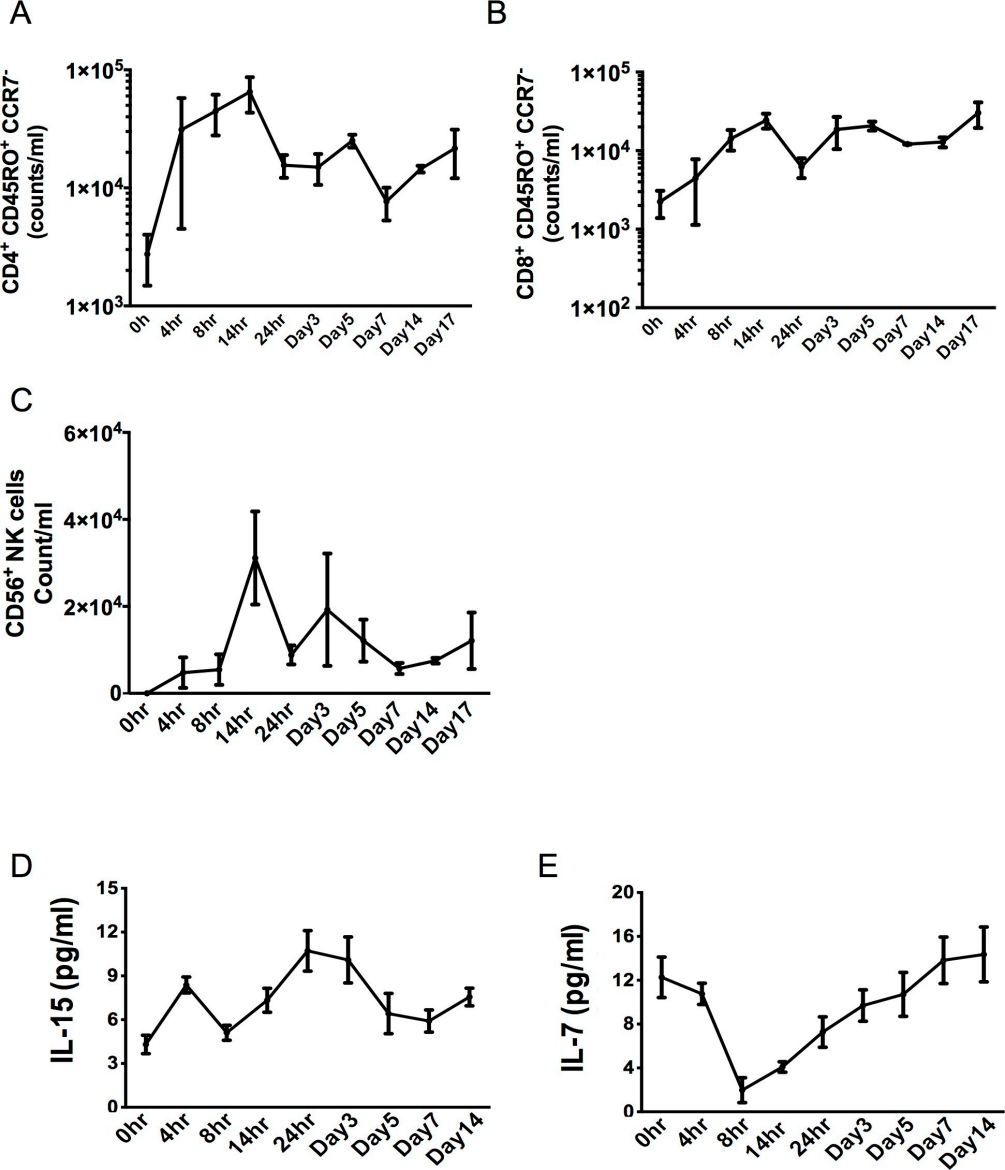
Post-resolution tissue accumulation of memory T cells and lymphocyte mitogens. Acute inflammation was triggered in the ventral aspect of forearm of healthy volunteers by the intradermal injection of 1.5 x 10^7^ UV-killed *E*. *coli* (UVKEc) suspended in 100 μl of sterile saline. A suction blister was raised over the inflamed site at the specified interval to collect the inflammatory exudate. Exudate was centrifuged to separate cells from the supernatant. The immune cell subsets were identified by polychromatic flow cytometry. The numbers of CD4^+^/CD45RO^+^/CCR7^-^ **(A),** CD8^+^/CD45RO^+^/CCR7^-^ memory T cells **(B)** and CD56^+^ NK cells **(C)** at the specified interval are shown here. IL-15 **(D)** and IL-7 **(E)** in the cell free exudate were measured using multiplex ELISA and their concentrations in the exudate at the specified intervals are shown here. n = 3 for each time point. Data presented as mean ± SEM.

Despite the resolution of acute inflammation, at least in line with the classical definition of antigen, granulocyte and cytokine clearance, we observed an increase in numbers of CD4^+^/CD45RO^+^/CCR7^-^ **(Fig 2A)** and CD8^+^/CD45RO^+^/CCR7^-^ memory T cells **(Fig 2B)** accumulating in the post-resolved tissue from D7; a trend towards a post-resolution increase in CD56^+^ NK cells was also detected, **Fig 2C**. This increase in T cells was associated with an equivalent increase in the T cell mitogens IL-15 as well as IL-7, **Fig 2D and 2E.**

**Fig 3:**
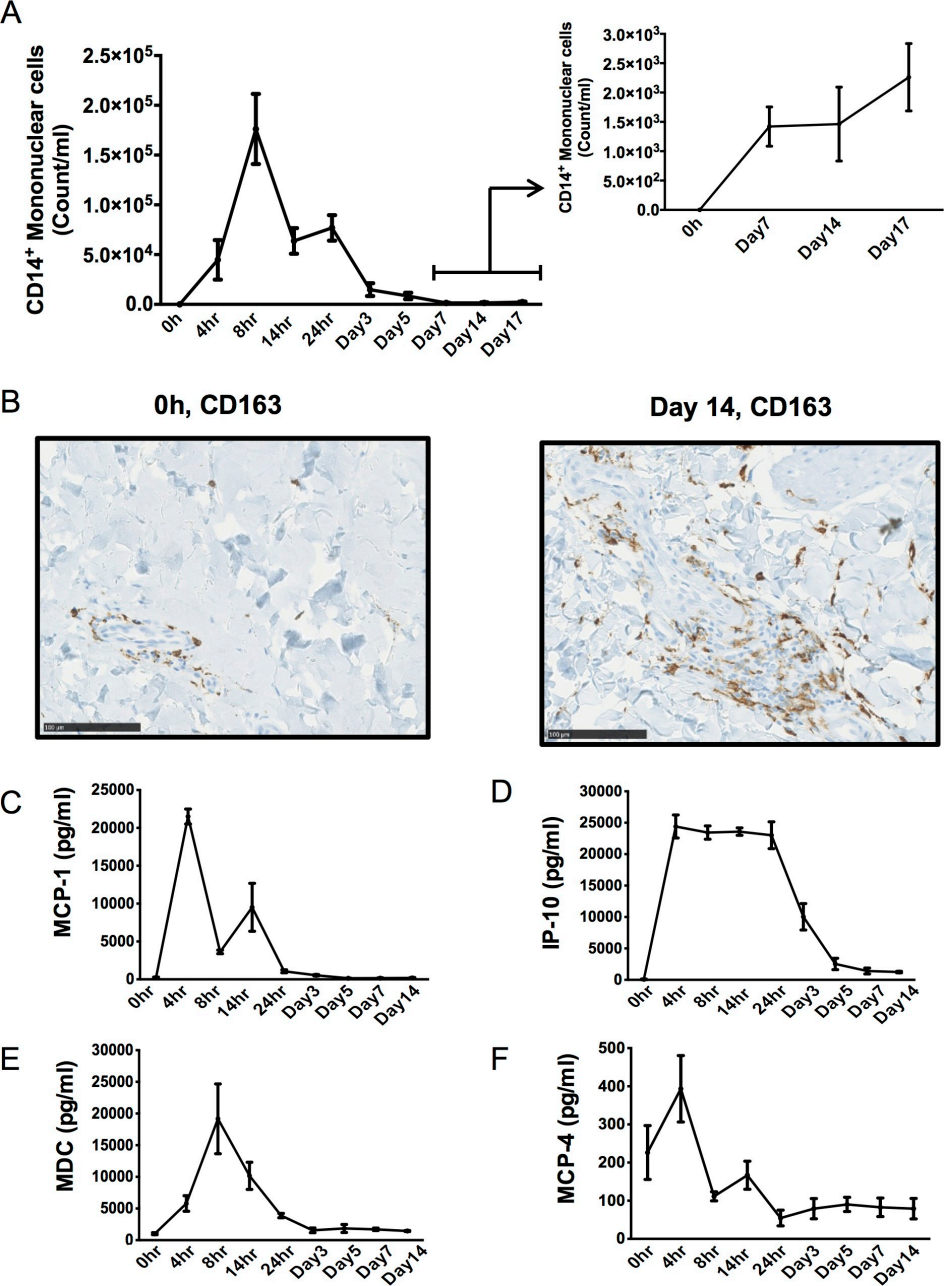
Post-resolution tissue accumulation of macrophages. Acute inflammation was triggered in the ventral aspect of forearm of healthy volunteers by the intradermal injection of 1.5 x 10^7^ UV-killed *E*. *coli* (UVKEc) suspended in 100 μl of sterile saline. A suction blister was raised over the inflamed site at the specified interval to collect the inflammatory exudate. Exudate was centrifuged to separate cells from the supernatant. The immune cell subsets were identified by polychromatic flow cytometry. The numbers of CD14^+^ mononuclear cells at the specified interval are shown here **(A)**. A 3mm skin punch biopsy was taken from the inflamed site under local anaesthesia at the specified interval and the formalin fixed paraffin embedded (FFPE) skin sections were probed by immunohistochemistry for CD163. The representative sections are shown here **(B).** MCP-1, IP-10, MDC and MCP-4 **(panels C-F)** in the cell free exudate were measured using multiplex ELISA and their concentrations in the exudate at the specified intervals are shown here **(C)**. n = 3 for each time point. Data presented as mean ± SEM.

In addition, polychromatic flow cytometry analysis of blister cells showed an initial peak in numbers of CD14^+^/HLA-DR^+^ monocytes/macrophages at 8 h and then again at 24 h post UVkEc injection, **Fig 3A**. However, closer analysis of these data up to day 14 suggested a potential increase in macrophages long after inflammation resolved. Indeed, this accumulation was more apparent when 3mm punch biopsies were carried out at day 14, which revealed a discrete population of perivascular CD163^+^ macrophages that were greater in number when compared to naïve skin tissue, **Fig 3B**. Measuring soluble mediators that control macrophage trafficking and function in the elicited blister fluid demonstrated a biphasic profile in MCP-1 **(Fig 3C)** mirroring that of macrophage kinetics during the first three days of the response, **Fig 3A.** We also found increases in IP-10, MDC and MCP-4 (**Fig 3D–3F)** during the acute phase of the response with levels MCP-4 remaining around 100pg/ml up to day 14.

### Post-resolution lipid mediator biosynthesis

**Fig 4:**
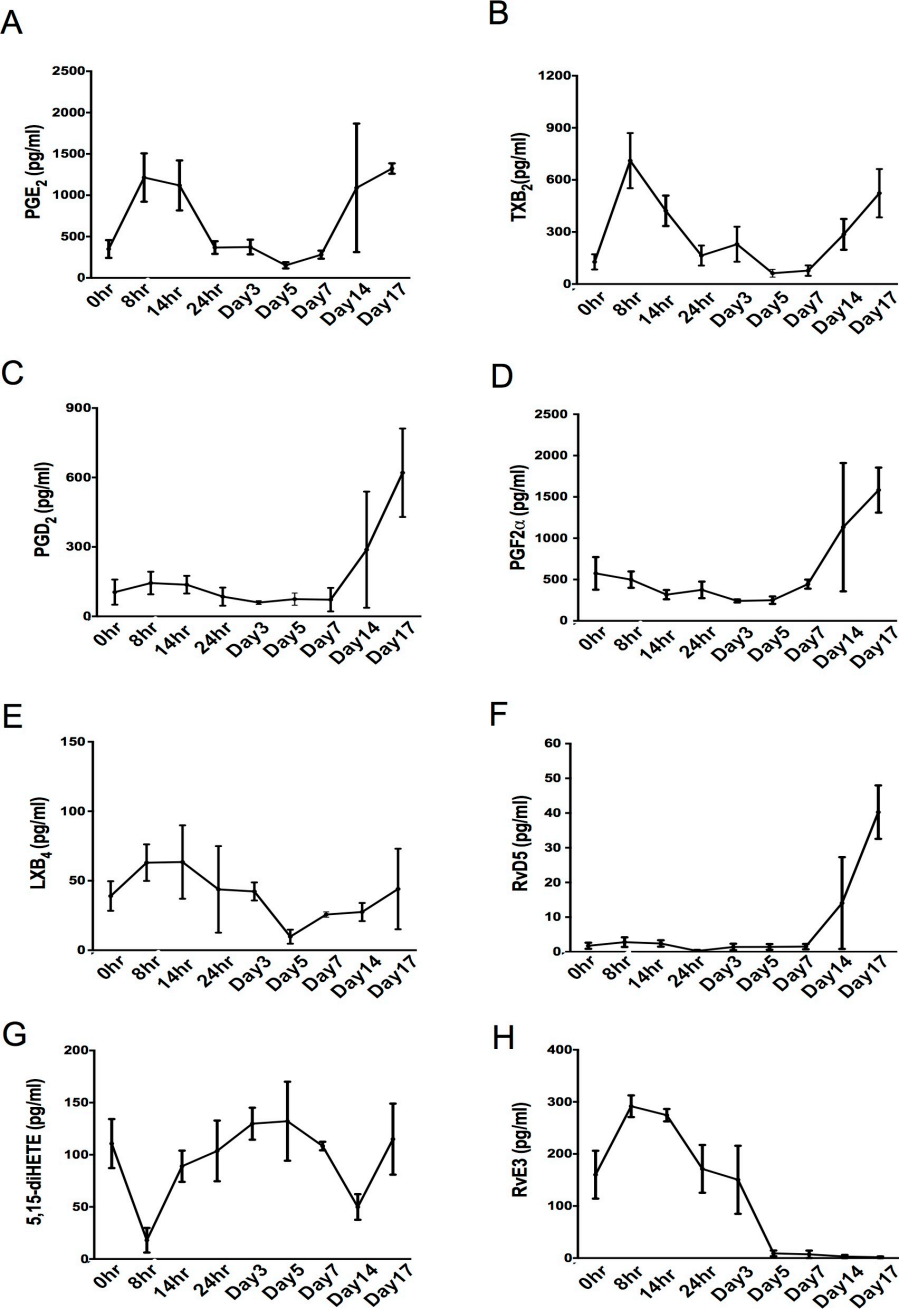
Increased lipid mediator biosynthesis during post-resolution biology. Acute inflammation was triggered in the ventral aspect of forearm of healthy volunteers by the intradermal injection of 1.5 x 10^7^ UV-killed *E*. *coli* (UVKEc) suspended in 100 μl of sterile saline. A suction blister was raised over the inflamed site at the specified interval to collect the inflammatory exudate. Lipid mediators in the cell free exudate were analysed by liquid chromatography tandem mass spectrophotometry (LC MS/MS). The levels of PGE_2_ **(A)**, TXB_2_ **(B),** PGD_2_ **(C),** PGF_2α_ **(D),** LXB_4_ **(E)**, 5,15-diHETE **(F)**, RvD5 **(G)**, 5,15 diHETE (G) and RvE3 **(H)** at specified interval are shown here. n = 3 for each time point. Data presented as mean ± SEM.

Using LC/MS-MS based profiling we identified from all three major bioactive metabolomes in elicited blister exudates including D-series resolvins and prostaglandins. These were identified in accordance with published criteria that include retention time and MS-MS spectra [14]. Quantitation of identified mediators in blister fluids revealed an expected increase in levels of PGE_2_ as well as TxB_2_ during the early onset phase (8h), which reduced back to baseline levels by 24 h post UVkEc injection, **Fig 4A and 4B**. However, from day 14-post UVkEc injection or 11 days post classical resolution we noted a second rise in these prostanoids as well as PGD_2_ and PGF_2α_, **Fig 4C and 4D**. Concentrations of pro-resolving mediators including resolvin D5 (RvD5), lipoxin B_4_ (LXB_4_), and the LX pathway marker 5,15 diHETE also increased at day 14. Whereas RvE3 concentrations were only elevated during the initial phases of the inflammatory response, demonstrating a differential regulation of lipid mediator biosynthesis at the site **Fig 4E–4H**.

### Post-resolution PGE_2_ maintains T cell numbers and proliferation

**Fig 5:**
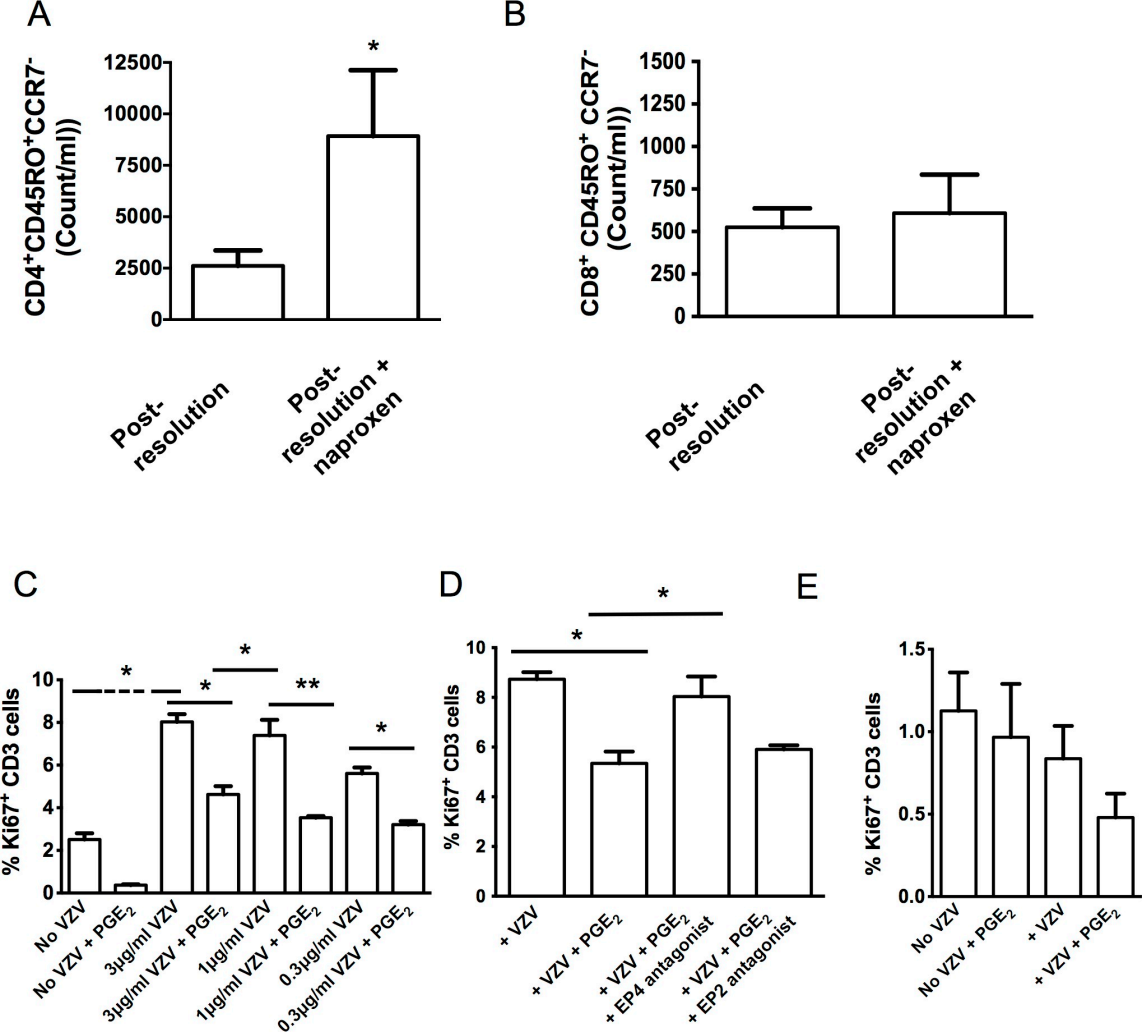
Inhibition of post-resolution prostanoids increases numbers of local memory CD4^+^ T cells. Acute inflammation was triggered in the ventral aspect of forearm of healthy volunteers by the intradermal injection of 1.5 x 10^7^ UV-killed *E*. *coli* (UVKEc) suspended in 100 μl of sterile saline. Inflammation was allowed to progress for 9 days after which volunteers were administered naproxen (500 mg, BD) orally for six days to block the rise in post-resolution prostanoids. On day 14 suction blister were elicited to enumerate cell profiles focusing on CD4^+^/CD45RO^+^/CCR7^-^ **(A)** and CD8^+^/CD45RO^+^/CCR7^-^ memory T cells **(B).** To determine what effect prostanoids have on memory T cell function, peripheral blood CD3^+^ cells were isolated from chicken pox-exposed individuals and incubated with varicella zoster antigen with/without PGE_2_ at concentrations found in post-resolution tissue **(C)** with/without EP 2/4 antagonists **(D)** while CD3^+^ cells from chicken pox naïve individuals were used as negative controls **(E)**. n = 3 for each time point. Data presented as mean ± se. *p≤0.05, **p≤0.01; as determined by ANOVA followed by Bonferroni t test or by two-tailed Student's t test.

To determine the pathophysiological relevance of these findings we dosed volunteers with the non-selective NSAID, naproxen, starting from day 9 post UVkEc injection and examined the site at day 14; our specific aim was to determine what role PGs play during post-resolution biology. We found that naproxen caused an increase in numbers of CD4^+^/CD45RO^+^/CCR7^-^ but not CD8^+^/CD45RO^+^/CCR7^-^ memory T cells, **Fig 5A and 5B**, respectively. Taking this further we isolated peripheral blood CD3^+^ T cells from chicken pox-exposed volunteers to determine the effects of PGE_2_ on memory T cell proliferation. Using concentrations of PGE_2_ found in post-resolution tissues, we found that this prostaglandin inhibited proliferation of VZV antigen-specific CD3^+^ T cells **(Fig 5C)** in an EP4 but not EP2 dependent manner, **Fig 5D**; CD3^+^ T cells from chicken pox naïve donors acted as a negative control, **Fig 5E**.

### Post-resolution PGE_2_ determines responses to secondary stimuli

**Fig 6:**
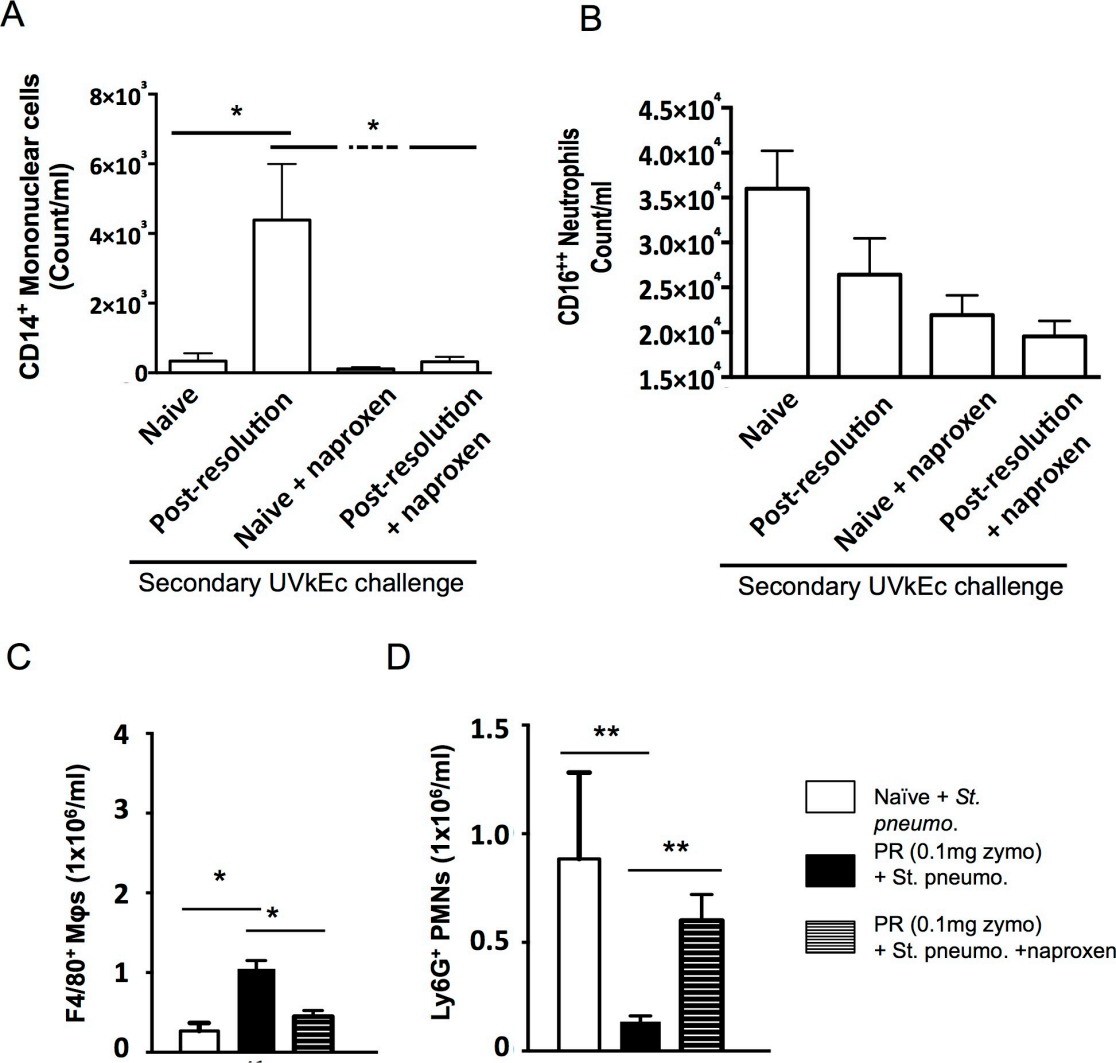
Post-resolution tissues mount a predominantly macrophage driven response that is cyclooxygenase mediated. Volunteers were injected intradermally with 1.5 x 10^7^ UV-killed *E*. *coli* (UVKEc) suspended in 100 μl of sterile saline in one forearm and 100μl of sterile saline only in the contralateral forearm. Inflammation was allowed to progress for 9 days after which one group of volunteers were administered naproxen (500 mg, BD) orally for six days to block the rise in post-resolution prostanoids and the other group remained untreated. On day 14, the forearm sites on all volunteers were re-injected with UVKEc and a suction blister was raised over the inflamed site at 4hr to collect inflammatory exudate. The number of mononuclear cells **(A)** and neutrophils **(B)** in the saline injected arm, untreated group (naive); UVKEc injected arm, untreated group (post-resolution); saline injected arm, naproxen treated group (naive + naproxen) and UVKEc injected arm, naproxen treated group (post-resolution + naproxen) are shown here. For mouse experiments **(panels C and D)**, 0.1 mg zymosan was injected in the peritoneum of male c57bl/6 mice. 14 days later either saline or live *S*. *pneumoniae* were injected i.p. and 4 h later the composition of the peritoneum was determined by polychromatic flow cytometry; naproxen was dosed from day 7. For human studies there were n = 3 volunteers for each group, while there were n = 5 mice for rodents studies/group. Data presented as mean ± se. *p≤0.05, **p≤0.01; as determined by ANOVA followed by Bonferroni t test or by two-tailed Student's t test.

Groups of volunteers were injected with either saline into one arm or UVkEc into the other. 14 days later UVkEc was injected into both sites—the original saline and UVkEc injected sites. Blister was raised at the site 4 hr after the secondary UVkEc challenge. The purpose of this experiment was to determine how tissues respond to secondary inflammatory stimuli following inflammatory resolution. Its transpires that tissue that experienced acute resolving inflammation elaborated substantially more CD14^+^ macrophages **(Fig 6A)** and comparatively fewer PMNs, **Fig 6B**. Repeating these experiments, but dosing volunteers with naproxen to inhibit the second post-resolution rise in prostanoids, revealed that this macrophage-dominated response was driven, in part, by cyclooxygenase-derive lipid mediators as numbers of CD14^+^ cells in inflammation experienced tissues were substantially reduced, **Fig 6A**; there was little effect on PMN numbers in these experiments.

These data challenge the idea that resolution leads tissue back to homeostasis, the physiological state they experienced before inflammation, but that events specific to the resolution response elicit a sequence of immunological events that control future responses to foreign and endogenous antigens and that are key in maintaining immune tolerance. Indeed, the composition of the post-resolution tissue in mice is immunologically similar to that seen in post-resolution human skin. Using zymosan model, we injected mice at day 14 (the time point of maximal lymphoid and myeloid infiltration as well as prostanoid synthesis) with *S*. *pneumonia* and determined the composition of the cavity 4h later. As with humans, post-inflamed murine tissues mounted a predominantly macrophage-driven response that was reversed with naproxen, **Fig 6C**. Unlike humans, however, numbers of PMNs were paradoxically elevated following cyclooxygenase inhibition in rodents, **Fig 6D**.

Therefore, acute inflammatory responses in both humans and rodents do not revert back to homeostasis but trigger a hitherto unappreciated sequence of immunological events that dictate subsequent immune response to infection.

## Discussion

In this report, we show that an i.d. injection of UV-killed *E*. *coli* in forearms of healthy male volunteers triggered a classical acute inflammatory flare as characterised by increased microvascular hyperaemia around the injection site. In addition, there was PMN infiltration, which peaked alongside cytokine/chemokine synthesis at 4 h with both of these acute inflammatory parameters along with vascular flow subsiding within 24–72 h. However, there was lag phase of a few days after which time we noted increased numbers of memory T cells as well as CD163^+^ macrophages within the post-resolved injection site as well as robust prostanoid and pro-resolving mediator (RvD5 and LXB_4_) biosynthesis. Its transpires that prostanoids control the numbers of memory CD4^+^ T during this post-resolution phase as dosing volunteers with a COX inhibitor, naproxen, caused an increase in memory CD4^+^ T cells numbers within the skin; findings that were verified *in vitro* to be through the EP2 receptor. Indeed, re-introducing a second stimulus back into the post-resolved tissue result in an increase in macrophage numbers compared to responses expected from naïve tissues injected with the same inoculum. In contrast, there were comparatively fewer PMNs; these effects were partially prostanoid mediated. Collectively, these data show that in healthy male human volunteers resolution of acute inflammation does not revert back to homeostasis, but triggers a prolonged phase of discreet immunological activity that affects responses to subsequent infection. We describe these processes as “*resolution leading to adapted homeostasis*” and most likely has a role in maintaining immune tolerance.

There is also evidence of immune dysfunction leading to chronic disease occurring long after clearance of the infectious stimulus. For instance, in a murine model of Sendai-induced para-influenza, despite clearing the infection mice progressed to develop an asthma-like disease mediated by sustained activity of natural killer T cells driving macrophages to produce interleukin-13 [11]. More recently, mice that received a single inoculum of *Yersinia pseudotuberculosis* experienced immune disruption in the gut weeks after bacterial clearance [12]. This disruption was characterised by lymphatic leakage in the mesenteric adipose tissue that redirected dendritic cells to the adipose compartment thereby preventing their proper accumulation in the mesenteric lymph node. Consequently, mucosal immune functions, including tolerance and protective immunity, were persistently compromised. Thus, even if the inciting stimulus is cleared, there is evidence of local “immunological mal-adaption”, predisposing tissues to chronic inflammation occurring months or years after the initial exposure; at least in response to some infections. However, the nature of this post-inflammation, immune mal-adaptation is not clearly understood and further research is warranted in this area.

As a result of the established effects of NSAIDs on the inflammatory response, PGE_2_ has always been considered as pro-inflammatory. However, its role in the wider innate and adaptive immune response is far more complex. For instance, while PGE_2_ can promote the activation, maturation and migration of dendritic cells, it’s been widely demonstrated to suppress both innate and antigen-specific immunity at multiple molecular and cellular levels [15,16]. Indeed, its complex role in adapted immunity was further resolved when it was found that it can suppress the differentiation of functionally-competent Th1-inducing DCs[17]. In fact, it was recently reported that the resulting “PGE_2_-DC” was a myeloid-derived suppressor cells capable of suppressing CTL responses [18–21]. PGE_2_ also directly inhibits IL-2 and the responsiveness of T cells to IL-2 thereby suppressing the activation and expansion of antigen-specific T cells [22,23]. PGE_2_ and other cAMP-elevating agents also act on humoral immunity as it enhances immunoglobulin class switching [15] as well as V(D)J recombination activity in pre- B-cell lines [24]. Thus, PGE_2_ exerts multiple modulatory effects on innate and adaptive immunity with its predominant effect/s most likely being context dependent. PGE_2_ is also linked with promoting the formation of pro-resolving mediators, where in a cAMP dependent manner it upregulates the expression of 15-lipoxygenase type 1 in human leukocytes which is the initiating enzyme in the biosynthesis of resolvins, protectins and lipoxins [25,26].

In contrast, the role of thromboxane in immune responses is less well described. Nonetheless, as a result of its vasoconstrictive properties thromboxane negatively regulates the spread of bacterial infections as well as T cell proliferative responses [27–29]. More recently, thromboxane has been shown to play a role in regulating the manner by which draining lymph nodes respond to vaccines and foreign antigens, playing a key role in the phenomenon of “lymph node shut down”. This is where, in steady state, the rate of lymph flow and output of cells in the efferent lymph is constant, but within hours of antigen or adjuvant injection there is a transitory decrease of lymphocytes exiting the efferent lymph [30–32]. Lymph node shutdown is an event thought to facilitate the development of adaptive immunity. Lipid mediators, including thromboxane, are thought to be the critical component in this process [31,33]. Thus, it must be appreciated that the role of prostanoids in immunity stretches far beyond that of acute inflammation where they are implicated in driving heat, redness, swelling and pain. Indeed, with the recognition of this post-resolution phase of inflammation a new role in regulating immune tolerance will become more apparent in time.

While we have only taken these studies to day 17 post *E*. *coli* injection, we suspect from previous work [9] that this post-resolution phase leading to “adapted homeostasis” progresses for a number of weeks if not months. It is also surprising that the increased local immunological activity is clinically silent, specifically that despite elevation in factors like prostanoids, which are known to contribute to pain, oedema and redness, these cardinal signs of inflammation are not apparent on the skin surface. It is also remarkable that this immunologically modified tissue should react so differently to secondary stimuli in terms of the cellular response mounted, which was predominantly macrophage mediated.

In summary, we report a novel sequence of events specific to resolution of acute inflammation in healthy humans, leading to “adapted homeostasis” that we propose might be essential for the maintenance of immune tolerance to endogenous antigens. We suspect that post-resolution prostanoid biosynthesis act as an internal checkpoint central to preventing the development of diseases driven by autoimmunity.
